# Functional ultrasound imaging and prewhitening analysis reveal MK-801-induced disruption of brain network connectivity

**DOI:** 10.3389/fphar.2025.1562102

**Published:** 2025-06-03

**Authors:** Erik Hakopian, Argishti E. Stepanian, Shan Zhong, Kofi A. Agyeman, Nancy Zepeda, Kevin Wu, Charles Liu, Darrin J. Lee, Vassilios Christopoulos

**Affiliations:** ^1^ Department of Neuroscience, University of California Riverside, Riverside, CA, United States; ^2^ Department of Biomedical Engineering, University of Southern California, Los Angeles, CA, United States; ^3^ Department of Bioengineering, University of California Riverside, Riverside, CA, United States; ^4^ Department of Neurological Surgery, Keck School of Medicine, University of Southern California, Los Angeles, CA, United States; ^5^ Neurorestoration Center, Keck School of Medicine, University of Southern California, Los Angeles, CA, United States; ^6^ Rancho Los Amigos National Rehabilitation Center, Downey, CA, United States

**Keywords:** functional ultrasound imaging (fUSI), brain network connectivity, prewhitening analysis, MK-801, NMDAR inhibition, memory, learning, neuropsychiatric disorders

## Abstract

**Background:**

Disruption of N-methyl-D-aspartate receptor (NMDAR) activity within the septohippocampal network - a critical circuit that includes the hippocampus, medial prefrontal cortex (mPFC) and other nuclei - is believed to contribute to learning and memory impairments. Although animal models using the NMDAR antagonist Dizocilpine (MK-801) replicate cognitive deficits associated with memory and learning disorders, the direct effects of MK-801 on brain network connectivity have not been well characterized.

**Objective:**

This study aims to explore the effects of MK-801 on brain network connectivity using functional ultrasound imaging (fUSI) and apply time series analysis methods to mitigate potential statistical confounds in functional connectivity assessments.

**Methods:**

fUSI was employed to assess changes in cerebral blood volume (CBV) and network connectivity in MK-801-treated mice. To account for the nonstationarity and autocorrelation inherent in fUSI time series, an AutoRegressive Integrated Moving Average (ARIMA) model was applied to stabilize the mean and remove autocorrelation, ensuring more reliable signal analysis.

**Results:**

Our analysis revealed that MK-801 significantly disrupts functional connectivity (FC) across key brain regions, including the hippocampus, mPFC, and striatum. We also demonstrated that removing autocorrelation from the fUSI time series mitigates the risk of spurious associations, enhancing the reliability of network analysis.

**Conclusion:**

This study demonstrates the importance of accounting for nonstationarity in fUSI time series to improve the accuracy of brain network connectivity analysis. Our findings indicate that MK-801-induced NMDAR inhibition disrupts connectivity both within and outside the septohippocampal circuit, offering new insights into the neural mechanisms underlying cognitive deficits in disorders affecting memory and learning.

## 1 Introduction

Cognitive deficits in learning and memory are common features of various neuropsychiatric and neurological disorders. One prominent hypothesis links these impairments to reduced activity of the N-methyl-D-aspartate receptor (NMDAR) (Lee and Zhou, 2019; Krystal et al., 1994; Newcomer et al., 1999; Clayton et al., 2002; Liu et al., 2019). Dizocilpine (MK-801), a potent non-competitive NMDAR antagonist, is widely used in animal studies to investigate memory and learning disorders and cognitive dysfunction (McLamb et al., 1990; Pitkänen et al., 1995; van der Staay et al., 2011; Wiescholleck and Manahan-Vaughan, 2012). By binding within the NMDAR channel and inhibiting calcium ion influx, MK-801 disrupts NMDA receptor-mediated neurotransmission, leading to transient deficits in learning, memory, and other cognitive processes.

The effects of MK-801 extend to specific neural circuits, particularly the septohippocampal network (Crown et al., 2024). This network, which includes the hippocampus, medial prefrontal cortex (mPFC), septum, cholinergic and GABAergic projections from the septal area to the hippocampus, plays an important role in learning and memory processes (Khakpai et al., 2013a). By blocking NMDAR activity, MK-801 disrupts normal synaptic transmission between the medial septum and hippocampus, impairing theta oscillations that are essential for hippocampal function (Abad-Perez et al., 2023). Additionally, NMDAR inhibition alters inhibition alters neurotransmitter levels, including acetylcholine and GABA, further exacerbating deficits in spatial learning and memory (Gonzalez et al., 1994). Perturbations in NMDAR function within the septohippocampal network have been implicated in cognitive dysfunctions associated with various neuropsychiatric disorders (Jodo, 2013).

Given the critical role of the septohippocampal network in cognitive functions and its sensitivity to NMDAR modulation, this network provides an ideal model for exploring the neural mechanisms underlying cognitive impairments linked to NMDAR dysfunction (Khakpai et al., 2013b). Recently, we showed that intraperitoneal (i.p.) injection of MK-801 in mice significantly reduces hemodynamic signals of areas within (i.e., hippocampus and mPFC) and outside the septohippocampal network (Crown et al., 2024). These findings align with clinical studies showing reduced neural activity in the hippocampus and prefrontal cortex of patients with schizophrenia (MacDonald et al., 2006; Gur and Gur, 2010; Bedford et al., 2012).

Building on our previous study, we examined the effects of MK-801 on functional connectivity (FC) between brain regions within and outside the septohippocampal network using functional ultrasound imaging (fUSI) technology (Tanter and Fink, 2014; Macé et al., 2011). fUSI visualizes neural activity by mapping local changes in cerebral blood volume (CBV), which are indirectly linked to neuronal activity through neurovascular coupling. This modality offers high resolution, broad spatial coverage, and exceptional sensitivity, enabling the capture of dynamic brain processes with precision. In this study, we made two key contributions: (1) we demonstrated that MK-801 disrupts dynamic FC among key brain regions, including the mPFC, hippocampus, and striatum, which are critical for memory, learning, and higher-order cognitive functions and (2) we introduced the pre-whitening analysis on fUSI data–an approach that removes autocorrelation and temporal dependencies from time series to ensure statistical validity–illustrating the importance of rendering data stationary before assessing FC. We demonstrated that, without prewhitening, fUSI time series of recorded regions of interest (ROIs) exhibit strong autocorrelation and non-stationarity, violating fundamental assumptions of correlation analysis and potentially misrepresenting the true associations between the ROIs. Overall, the results showed that MK-801 not only reduces the activity of brain regions within and outside the septohippocampal network, but also disrupts the FC between them as a function of time.

## 2 Materials and methods

### 2.1 Animal acquisition and surgical procedures

Sixteen male C57BL/6 mice, aged between 8 and 12 weeks, were procured from Charles River Laboratories (Hollister, CA) and stratified into two principal experimental cohorts: a vehicle control group receiving saline administration (n = 8), and a treatment group receiving MK-801 (n = 8; 1.5 mg/kg). Prior to experimental procedures, the mice were anesthetized with 5% isoflurane, delivered in a carrier gas mixture comprised of oxygen and nitrous oxide in a 1:2 ratio and then maintained at a constant rate (1.5%–2%) through surgery and data acquisition. Body temperature was regulated throughout recordings by placing animals on an electric heating pad. Hair on the cranial region of each mouse was removed using a commercially available depilatory cream (Nair, Pharmapacks). All procedures were approved by the University of Southern California, Institutional Animal Care and Use Committee (IACUC #21006).

### 2.2 Data acquisition

**FIGURE 1:**
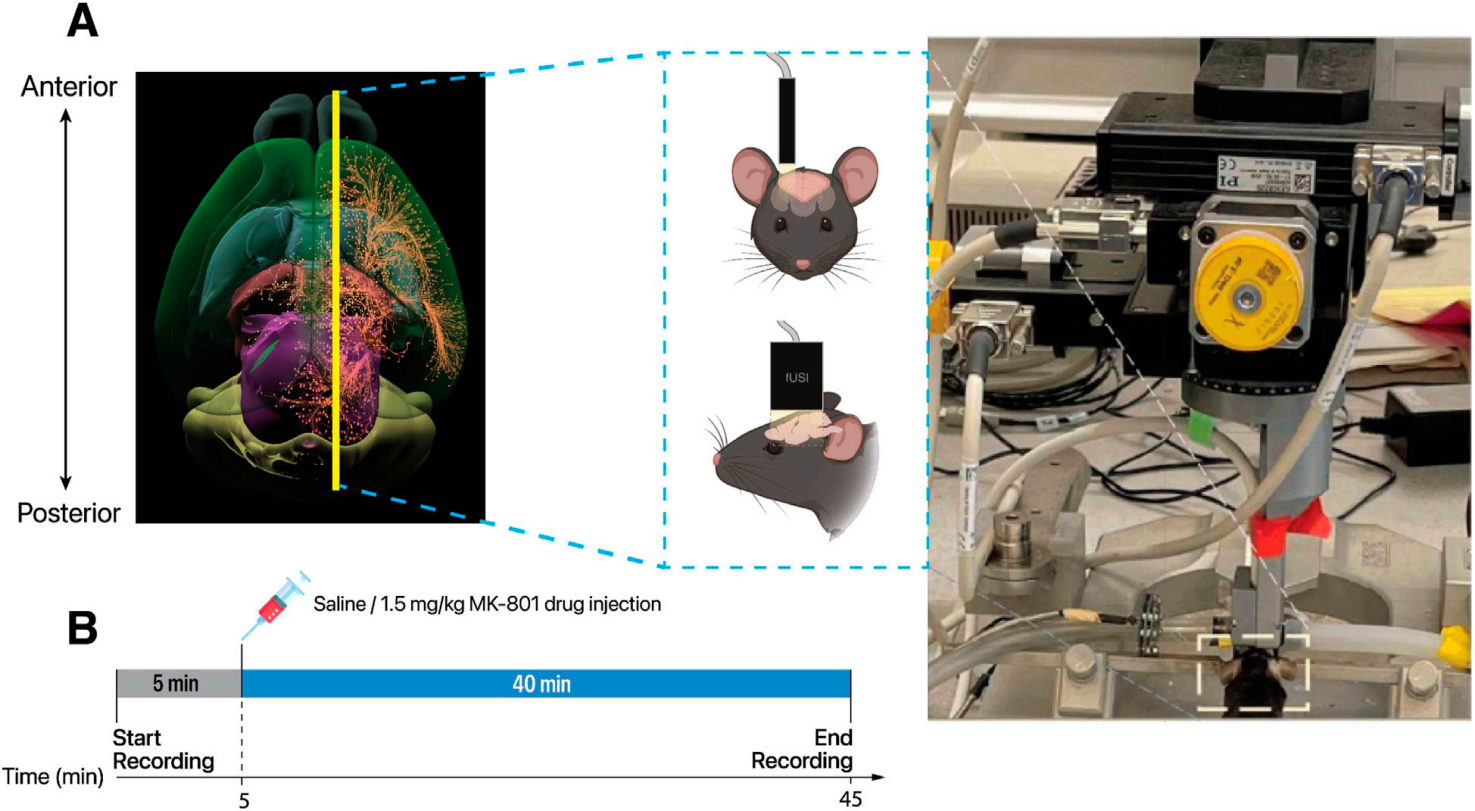
Experimental setup for investigating the effects of MK-801 on functional connectivity in the mouse brain using fUSI. **(A)** Top-down view of the mouse brain illustration with the yellow line indicating the sagittal imaging plane used for data acquisition. The diagram includes a schematic of the ultrasound probe placement on the mouse skull and the experimental setup, featuring an anesthetized mouse secured in a stereotaxic frame under the Iconeus One motorized system. **(B)** Timeline of the experimental protocol. Following a 5-minute baseline recording, mice received an i.p. injection of either saline (control) or MK-801 (1.5 mg/kg). fUSI data were continuously acquired for an additional 40 minutes post-injection.

**FIGURE 2:**
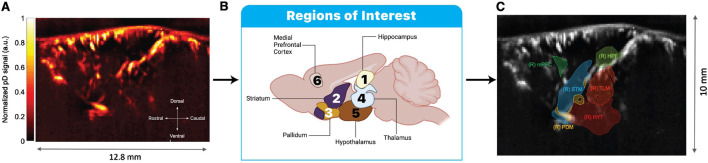
Plane selection and ROI extraction for fUSI analysis. **(A)** Representative power Doppler (pD) image showing the normalized signal intensity across the sagittal imaging plane. **(B)** Schematic illustration of the six ROIs analyzed in this study: hippocampus (1), striatum (2), pallidum (3), thalamus (4), hypothalamus (5), and medial prefrontal cortex (mPFC) (6). **(C)** ROI masks overlaid on the pD image as defined through the Iconeus One system’s automatic region selection tool. Acronyms in the image represent: (R) HPF: right hippocampal formation; (R) mPFC: right medial prefrontal cortex; (R) STR: right striatum; (R) TLM: right thalamus; (R) HYT: right hypothalamus; and (R) PDM: right pallidum. The different colors of the ROI masks are randomly assigned by the software solely to visually distinguish between adjacent brain regions and have no functional significance. Scale bar indicates 10 mm in the dorsal-ventral axis.

Power Doppler (pD) images were obtained using the Iconeus One scanner (Iconeus, Paris, France). Image acquisition was performed using a linear ultrasound transducer array (fUSI probe) with 128 channels, operating at a center frequency of 15.6 MHz and a pitch of 0.1 mm. This methodology enables a large field of view (12.8 mm width, 10 mm depth and 400 µm plane thickness) with a spatial resolution of 100 µm × 100 µm in-plane. The transducer was mounted on a motorized system and placed on the intact skull and skin along a sagittal plane on the right side to image areas within and outside the septohippocampal network (Figure 1A). A typical pD vascular map from a representative animal is illustrated in Figure 2A. Within this sagittal plane, we recorded activity from six distinct ROIs: hippocampus, mPFC, striatum, thalamus, hypothalamus and pallidum (Figure 2B). Note that other regions that are part of the septohippocampal network, such as the medial septal nucleus (MSN) were not recorded, since they were not accessible from the selected 2D imaging plane. The experimental protocol consisted of a 5-min baseline recording, followed by an i. p. injection of either 0.2 cc of saline or 1.5 mg/kg of MK-801 (Figure 1B). The i. p. injection was administered using a butterfly needle. To minimize the risk of motion artifacts from the injection, the butterfly needle was inserted prior to the recording session. Images were recorded uninterrupted for an additional 40 min post-injection, allowing us to capture dynamic CBV changes in response to drug administration. This approach was informed by previous studies showing that the concentration of MK-801 in the brain peaks approximately 40–60 min post-injection Wegener et al. (2011).

### 2.3 Plane selection and imaging

The target image plane was determined by co-registering a 3D fUSI whole-brain image of each mouse to a standard Allen Mouse Brain Common Coordinate Framework brain atlas utilizing dedicated software available with the Iconeus One scanner. This enabled the identification and extraction of the 6 ROIs (Figure 2C). The probe was fixed steadily throughout experiments on a motorized system with the field of view transverse and intersecting the co-registered sagittal plane. The imaging data were acquired through the compounding of 200 frames, captured at a frame rate of 500 Hz. This process employed 11 tilted plane waves, incrementally separated by 2°, spanning from −10° to +10°. The acquisition sequence was executed at a pulse repetition frequency (PRF) of 5.5 kHz, employing real-time continuous acquisition of successive blocks. Each block consisted of 400 ms of compounded plane wave images, followed by a 600 ms pause between pulses.

### 2.4 Data pre-processing

The Iconeus One acquisition system generated pD images pre-processed with built-in phase-correlation based sub-pixel motion registration and singular-value-decomposition (SVD) based clutter filtering algorithms Ledoux et al. (1997). These algorithms were used to separate tissue signal from blood signal to obtain pD images. Potential physiological and motion artifacts were addressed through the adoption of rigid motion correction techniques, which have proven effective in fUSI and other neuroimaging modalities Stringer and Pachitariu (2019). These motion correction techniques were integrated with high-frequency filtering algorithms to suppress noise-related artifacts. Specifically, a low-pass filter with a normalized passband frequency of 0.02 Hz and a stopband attenuation of 60 dB was implemented. This filter incorporated a delay compensation mechanism to mitigate any temporal distortions introduced by the filtering process itself, thereby facilitating the effective removal of high-frequency fluctuations from the pD signal data.

### 2.5 Statistical analysis tools

All analysis was performed using MATLAB 9.12.0.1927505 (R2022a) to 24.1.0.2537033 (R2024). We first calculated the normalized percent change in CBV ( Δ CBV) relative to baseline measurements—defined as the average signal during the initial 5 min prior to saline or MK-801 injection—for the time series of each ROI. To evaluate the effects of treatment (MK-801 vs. saline), ROIs, and time on Δ CBV, we performed a three-way mixed analysis of variance (ANOVA). Treatment (MK-801 vs. saline) served as the between-subject factor, while time and brain regions (6 ROIs) served as the within-subjects factor. For the time factor, we compared the mean Δ CBV values from two distinct periods: the baseline period (average of first 5 min pre-injection) and the final period (average of last 5 min of the 40-min post-injection recording) for each ROI in each animal. This analysis was conducted using data from eight animals per treatment group.

To further elucidate the magnitude of the drug effects between different ROIs, we performed a *post hoc* pairwise t-test analysis comparing baseline (0–5 min) and final period (40–45 min) of recordings for each ROI in both treatment groups. Statistical significance was assessed using paired t-tests (two-tailed, α=0.05 ) for each ROI. To control for multiple comparisons, we implemented the Benjamini–Hochberg (BH) false discovery rate (FDR) correction procedure. For each experimental group, p-values were ordered from lowest to highest, with each p-value assigned a rank (i) . The critical threshold for significance at each rank was calculated according to Equation 1.

$${\text{critical threshold}}_{i}=\frac{i}{n}\times \alpha$$ (1)

Where n represents the total number of comparisons (6 ROIs) and α=0.05 .

Subsequently, we performed a dynamic FC analysis between the six recorded ROIs for the two groups of animals. To characterize the temporal evolution of FC patterns, we implemented a two-stage analytical approach. First, we divided the 45-min recording period into nine non-overlapping 5-min windows to visualize the progressive changes in network FC [i.e., rolling functional connectivity (RFC)] over time (Figure 5). For each window, we computed the Pearson correlation matrices between all ROI pairs for each animal, providing a snapshot of the network dynamics at different time intervals. Prior to averaging across animals, we applied the Fisher r-to-Z transformation - Z=0.5×ln((1+r)/(1−r)) - to each correlation coefficient to normalize their distribution. The final correlation matrix for each window was obtained by averaging these Z-transformed values across all eight animals within each experimental group and then converting the results back to the correlation domain via the inverse Fisher transformation.

In addition to the 5-min window analysis, we implemented an RFC approach with finer temporal resolution to quantitatively assess temporal changes in functional connectivity. This approach used 60-s non-overlapping windows to calculate the Pearson correlation coefficient r(t) between pairs of prewhitened time series across the entire recording duration (2,700 s), Equation 2.

$$r\left(k\right)=\frac{\sum_{i=t_{k}}^{t_{k}+\Delta t}\left(x\left(i\right)-{\bar{x}}_{k}\right)\left(y\left(i\right)-{\bar{y}}_{k}\right)}{\sqrt{\sum_{i=t_{k}}^{t_{k}+\Delta t}{\left(x\left(i\right)-{\bar{x}}_{k}\right)}^{2}\sum_{i=t_{k}}^{t_{k}+\Delta t}{\left(y\left(i\right)-{\bar{y}}_{k}\right)}^{2}}}$$ (2)

Where

• r(k) is the Pearson correlation coefficient at the k‐th 60-second window.
• $t_{k}$ is the start time of the k‐th window.
• Δt=60 seconds is the window duration.
• ${\bar{x}}_{k}$ and ${\bar{y}}_{k}$ are the mean values of x and y in the interval $\left[t_{k},t_{k}+\Delta t\right]$ .

Following the previous analytical approach, we applied the Fisher r-to-Z transformation to normalize the distribution of correlation coefficients. To characterize the trends in correlation changes over time, we fitted a linear regression model to the Z-transformed correlation values, allowing us to identify and quantify any significant patterns or progressive shifts in connectivity throughout the experiment. We chose non-overlapping windows to ensure statistical independence between observations, which is crucial for the validity of subsequent regression analyses.

This analysis provided eight slopes per ROI pair (corresponding to the eight animals) in each experimental group. We then conducted two-sample independent t-tests directly comparing the distributions of these slopes (MK-801 slopes versus saline slopes) for each ROI pair. This approach specifically tested whether the rate of connectivity change over time differed significantly between treatment groups for each pair of ROIs. The resulting t-scores provided a standardized measure of the differential effects of MK-801 versus saline on the trajectory of functional connectivity dynamics. Negative t-scores indicated a greater connectivity decrease in the MK-801 group compared to the saline group. To control for multiple comparisons across all ROI pairs, we applied BH procedure for FDR correction.

### 2.6 Prewhitening the pD time series

**FIGURE 3:**
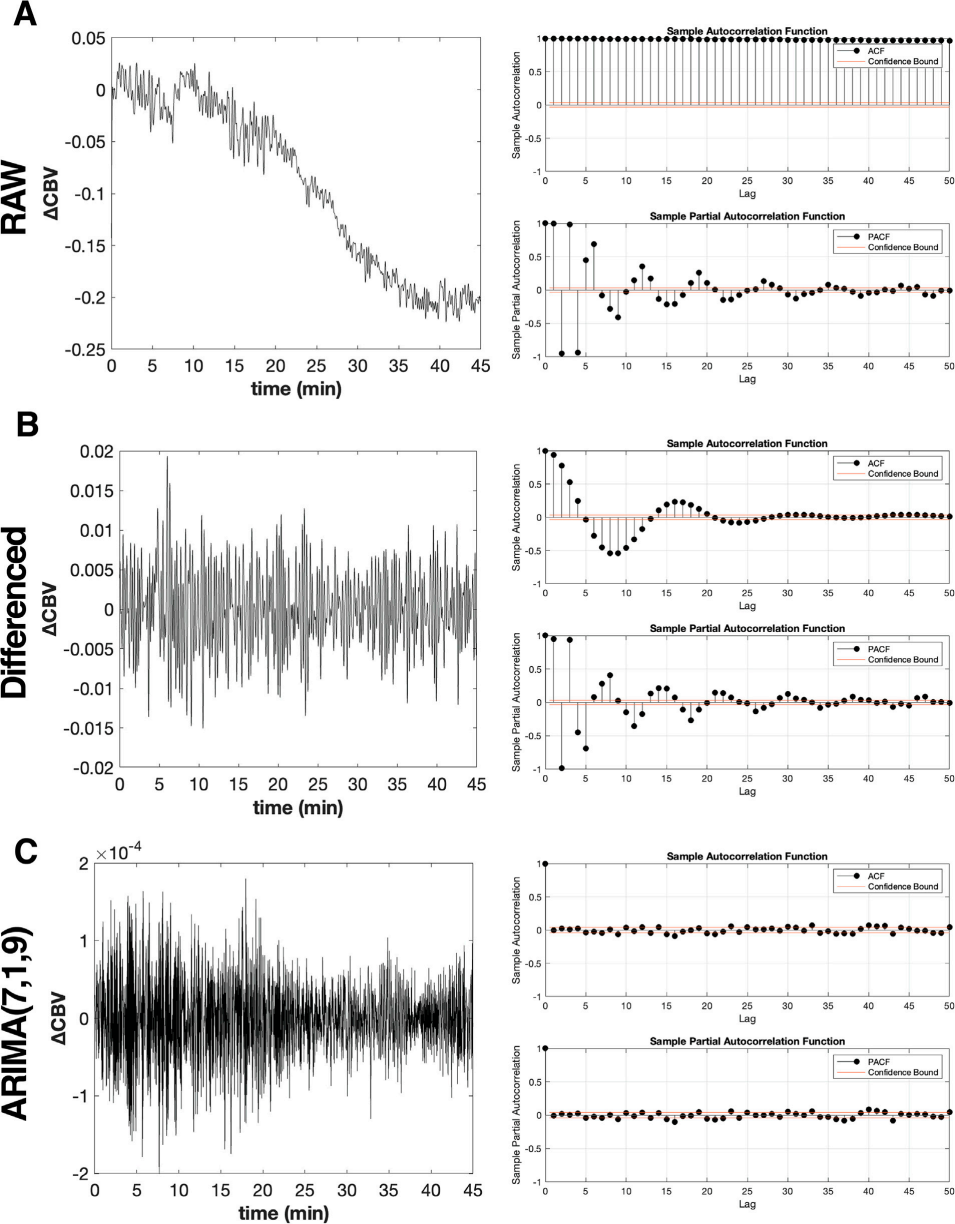
Transformation of non-stationary hippocampal CBV time series into stationary time series using ARIMA model. **(A)** Raw hippocampal CBV time series data from a representative MK-801-treated mouse (left panel), exhibiting non-stationary with respect to mean and variance pattern with an overall decreasing trend. The corresponding autocorrelation function (ACF) and partial auto-correlation function (PACF) plots (right panel) demonstrate significant autocorrelation across multiple lags, indicating strong temporal dependencies in the non-prewhitened data. **(B)** First-order differenced, ARIMA(0,1,0), hippocampal CBV time series data (left panel), showing improved stationarity with a more consistent mean and variance across the series. The ACF and PACF plots (right panel) reveal a reduction in autocorrelation compared to the raw data, but significant correlations still persist at certain lags. **(C)** ARIMA(7,1,9) prewhitened hippocampal CBV time series data (left panel), demonstrating a stationary process with constant mean (μ ≈ 0) and variance across the series. The corresponding ACF and PACF plots (right panel) show a dramatic reduction in autocorrelation, with the majority of lags falling within the confidence interval (red lines). This indicates that the ARIMA(7,1,9) model successfully captures and removes the temporal dependencies present in the raw hippocampal CBV time series data, resulting in a more stationary and uncorrelated process suitable for subsequent analyses.

Initial inspection of the motion-corrected pD time series from the six ROIs revealed pronounced nonstationarity in the mean and strong autocorrelation. This was demonstrated by plotting the CBV changes alongside the AutoCorrelation Function (ACF) and Partial AutoCorrelation Function (PACF) histograms for a representative ROI (i.e., hippocampus) from a typical animal (Figure 3A). To calculate the Pearson correlation coefficient r between time series, it is essential to ensure that individual series are stationary and non-autocorrelated (Box et al., 2015; Priestley, 1971; Granger and Newbold, 1974; Haugh, 1976). Ensuring stationarity and removing autocorrelation from the series is crucial for obtaining valid correlation results, as failing to do so may lead to spurious correlations that do not accurately reflect the true relationship between the time series. A notable example from econometrics illustrates the issue of spurious correlations, where a study initially reported spurious results Coen et al. (1969), which were later corrected using prewhitening techniques Box and Newbold (1971). Recently, in neuroscience, the problem of spurious correlations arising from nonstationary time series has gained recognition, pointing out the necessity of appropriate preprocessing methods (Cordes et al., 2001; Christova et al., 2011; Christopoulos et al., 2012b; Christopoulos et al., 2012a; Zalesky et al., 2014; Sakellaridi et al., 2015).

The solution to this issue, pioneered by researchers such as Box, Priestley, and Granger (Box et al., 1978; Priestley, 1971; Granger and Newbold, 1974), involves transforming each univariate time series into a stationary and non-autocorrelated form by appropriately modeling the series and extracting the residuals, also known as “innovations”. The correlation between the innovations represents the true relationship between two time series, free from spurious effects caused by nonstationarity and autocorrelation (Box et al., 1978; Jenkins and Watts, 1968; Granger and Newbold, 1974). This preprocessing step, known as “prewhitening”, typically involves fitting an AutoRegressive Integrated Moving Average (ARIMA) model Box et al. (2015). The ARIMA model combines three key components: AutoRegressive (AR), Integrated (I), and Moving Average (MA), represented by the parameters p , d , and q , respectively. This approach enables modeling complex time series by accounting for various temporal dependencies:

1. The AR (p) component models the dependency on past values.
2. The I (d) component achieves constant mean and variance through sequential differencing.
3. The MA (q) component accounts for the dependency on past forecast errors.

By fine-tuning these parameters, we can forecast future time points $\left(y_{t}\right)$ based on their preceding values $\left(y_{t-p}\right)$ . This process explicitly incorporates the autocorrelation structure present in the data. While ARIMA models are widely recognized as a standard tool for time series analysis, we include a brief overview here to clarify the specific methodology applied in our analysis.

#### 2.6.1 Integration (I)

Forecasting models, including ARIMA, rely on the fundamental assumption of statistical consistency in the input data, meaning that these models require the time series to exhibit constant mean and variance over time. To meet these criteria and stabilize a time series, we employ differencing, which involves calculating the changes between consecutive observations to remove trend and seasonality.

Notably, the “integrated” component d of the ARIMA model indicates the number of times the differencing operation must be applied to achieve consistency in the mean and variance of the time series. To verify the effectiveness of differencing, we used the Augmented Dickey-Fuller (ADF) test, which assesses the presence of a unit root in the differenced data. A non-significant result indicates the absence of a unit root, confirming that the series has reached the desired stationarity. This ensures that the data is now appropriate for the subsequent ARIMA modeling steps.

Figure 3B (left panel) shows the hippocampal Δ CBV for the representative animal after applying integration - i.e., differencing. The integration successfully removed the trend, centering the mean Δ CBV around zero while having constant variance throughout the series. However, while integration improved stationarity, it did not fully eliminate autocorrelation in the residuals. The ACF and PACF plots (Figure 3B, right panel) reveal a reduction in autocorrelation compared to the raw data (Figure 3A, right panel). Nevertheless, several statistically significant autocorrelations persist, particularly at the initial lags. This is especially important for our application, as we aim to evaluate synchronous correlations between ROIs (i.e., lag = 0).

#### 2.6.2 Autoregressive (AR) component

The AR component of the ARIMA model captures the dependency of the current observation on its past values. In an AR process, each data point is predicted as a linear combination of its previous observations, plus a random error term. The general form of an AR (p) model, where p is the order of the autoregressive process, can be expressed as:

$$\text{AR}\left(p\right):y_{t}=\omega +\phi_{1}y_{t-1}+\phi_{2}y_{t-2}+\cdots +\phi_{p}y_{t-p}+\varepsilon_{t}$$ (3)

Here, $y_{t}$ is the value of the series at time t , ω is the constant (intercept) term, $y_{t-1},y_{t-2},\ldots ,y_{t-p}$ are the lagged values of the series, $\phi_{1},\phi_{2},\ldots ,\phi_{p}$ are the weights of each lag, and $\varepsilon_{t}$ is the error term (white noise) at time t .

Selection of the appropriate order p is typically based on analyzing the PACF as it measures the direct correlation between an observation $y_{t}$ and a lag of itself $y_{t-k}$ , while controlling for the effects of intermediate lags. In practice, the PACF values at each lag correspond to the coefficients of AR models of increasing order (Equation 3), where each coefficient represents the direct effect of that lag when controlling for all shorter lags such that the PACF at lag k is equivalent to the coefficient $\phi_{k}$ in an AR (k) model. For example:

• PACF at lag 1 is $\phi_{1}$ in AR(1): $y_{t}=\omega +\phi_{1}y_{t-1}+\varepsilon_{t}$

• PACF at lag 2 is $\phi_{2}$ in AR(2): $y_{t}=\omega +\phi_{1}y_{t-1}+\phi_{2}y_{t-2}+\varepsilon_{t}$

• PACF at lag 3 is $\phi_{3}$ in AR(3): $y_{t}=\omega +\phi_{1}y_{t-1}+\phi_{2}y_{t-2}+\phi_{3}y_{t-3}+\varepsilon_{t}$

By identifying the lag at which PACF values become insignificant, we can estimate the appropriate order p for our AR model.

#### 2.6.3 Moving averages (MA) component

The moving average component is mathematically very similar to the AR model (Equation 3) such that:

$$MA\left(q\right):\;y_{t}=\omega +\theta_{1}\varepsilon_{t-1}+\theta_{2}\varepsilon_{t-2}+\cdots +\theta_{q}\varepsilon_{t-q}+\varepsilon_{t}$$ (4)

Where $y_{t}$ is the observed value at time t , ω is a constant term, $\theta_{1},\theta_{2},\ldots ,\theta_{q}$ are the MA coefficients at each lag, $\varepsilon_{t}$ is the error term at time t , and q is the order of the MA process. The key difference between Equation 3 and Equation 4 is that while the AR model predicts a time point based on weighted past observations, the MA model uses weighted past forecast errors to model the present time point. Hence, MA models the dependence on past unobserved white noise error terms, representing random shocks Christova et al. (2011). In the context of the fUSI analysis, MA can capture short-term fluctuations and measurement errors in CBV changes that are not explained by the overall trend or the AR component. This is particularly relevant for modeling transient changes in CBV that may not persist over longer time scales. Incorporating the MA component into the ARIMA model enables a more thorough representation of the temporal dynamics in FC, which can enhance the accuracy of the connectivity estimates.

#### 2.6.4 ARIMA model

After determining the appropriate integration, AR and MA processes, we combine Equation 3 and Equation 4 to construct the general ARIMA model. The comprehensive equation for the ARIMA model is expressed as:

$$y_{t}=\omega +\phi_{1}y_{t-1}+\cdots +\phi_{p}y_{t-p}+\theta_{1}\varepsilon_{t-1}+\cdots +\theta_{q}\varepsilon_{t-q}+\varepsilon_{t}$$ (5)

The ARIMA model encapsulates the autocorrelation structure inherent in the time series data (Equation 5). Consequently, we obtain the stationary and nonautocorrelated residuals (i.e., “innovations”) by subtracting the ARIMA model’s predictions from the original time series. This process can be expressed as a rearrangement of Equation 5:

$$\varepsilon_{t}=y_{t}-\left(\omega +\overset{p}{\underset{i=1}{\sum }}\phi_{i}y_{t-i}+\overset{q}{\underset{j=1}{\sum }}\theta_{j}\varepsilon_{t-j}\right)$$ (6)

where $\varepsilon_{t}$ represents the residuals at time t , $y_{t}$ is the original time series at time t , and the terms within the parentheses constitute the ARIMA model. Note that $\varepsilon_{t}$ is called “innovation” because it represents the unpredictable “shock” or “new information” at time t that cannot be inferred from past values of $y_{t}$ or past errors. It is assumed to be independently and identically distributed (i.i.d) with zero mean and constant variance, i.e., $\varepsilon_{t}∼N\left(0,\sigma^{2}\right)$

#### 2.6.5 Model selection

The identification of an appropriate ARIMA (p,d,q) model is crucial for effective prewhitening across multiple time series. To ensure consistency and facilitate valid comparisons in subsequent analyses, we aimed to identify a single ARIMA model that could be uniformly applied across all time series to effectively remove their autocorrelation structure. This decision aligns with common practices in neuroscience research aiming to minimize preprocessing variability that could complicate the interpretation of FC results (Christova et al., 2011; Christopoulos et al., 2012b; Sakellaridi et al., 2015).

For each time series, we first identified multiple candidate ARIMA models by calculating the Bayesian Information Criterion (BIC) for various combinations of autoregressive (p) and moving average (q) orders. Having observed that a differencing order of d = 1 successfully achieved statistical consistency (explained in section 2.6.1) across all time series, we maintained this integration order throughout the model selection process. The range of p and q and values was determined individually for each time series by observing the cutoff of significant lags in their respective PACF and ACF plots (see Sections 2.6.2, 2.6.3 for details on AR and MA order selection, respectively). For each time series, we retained the three ARIMA specifications yielding the lowest BIC scores.

These candidate models were then systematically evaluated across all time series to identify a single specification that could effectively remove autocorrelation throughout the entire dataset. The effectiveness of autocorrelation removal was assessed using critical bounds defined by Equation 7.

$$\pm \frac{1.96}{\sqrt{\left(T-d\right)}}$$ (7)

where T represents the length of the time series and d is the degree of differencing. A time lag was considered to have non-significant autocorrelation if it fell within these bounds. For each candidate model, we computed the total number of lags across all time series that became insignificant after applying the ARIMA transformation. This quantitative assessment was combined with visual inspection of the resulting ACF and PACF plots to evaluate the effectiveness of autocorrelation removal.

Through this comprehensive evaluation process, we identified ARIMA(7,1,9) as the specification that consistently reduced autocorrelation across all time series while maintaining uniform transformation properties. Using this model, we computed the residuals for each time series according to Equation 6. The effectiveness of this model is demonstrated in Figure 3C, using the representative hippocampal CBV time series, which shows both stationary behavior in the transformed time series (left panel) and successful removal of autocorrelation as evidenced by the ACF and PACF plots (right panel). This unified approach ensured that subsequent correlation analyses would be conducted on comparably transformed data, maintaining the validity of our FC assessments.

## 3 Results

### 3.1 MK-801 reduces cerebral blood volume across multiple brain regions

**FIGURE 4:**
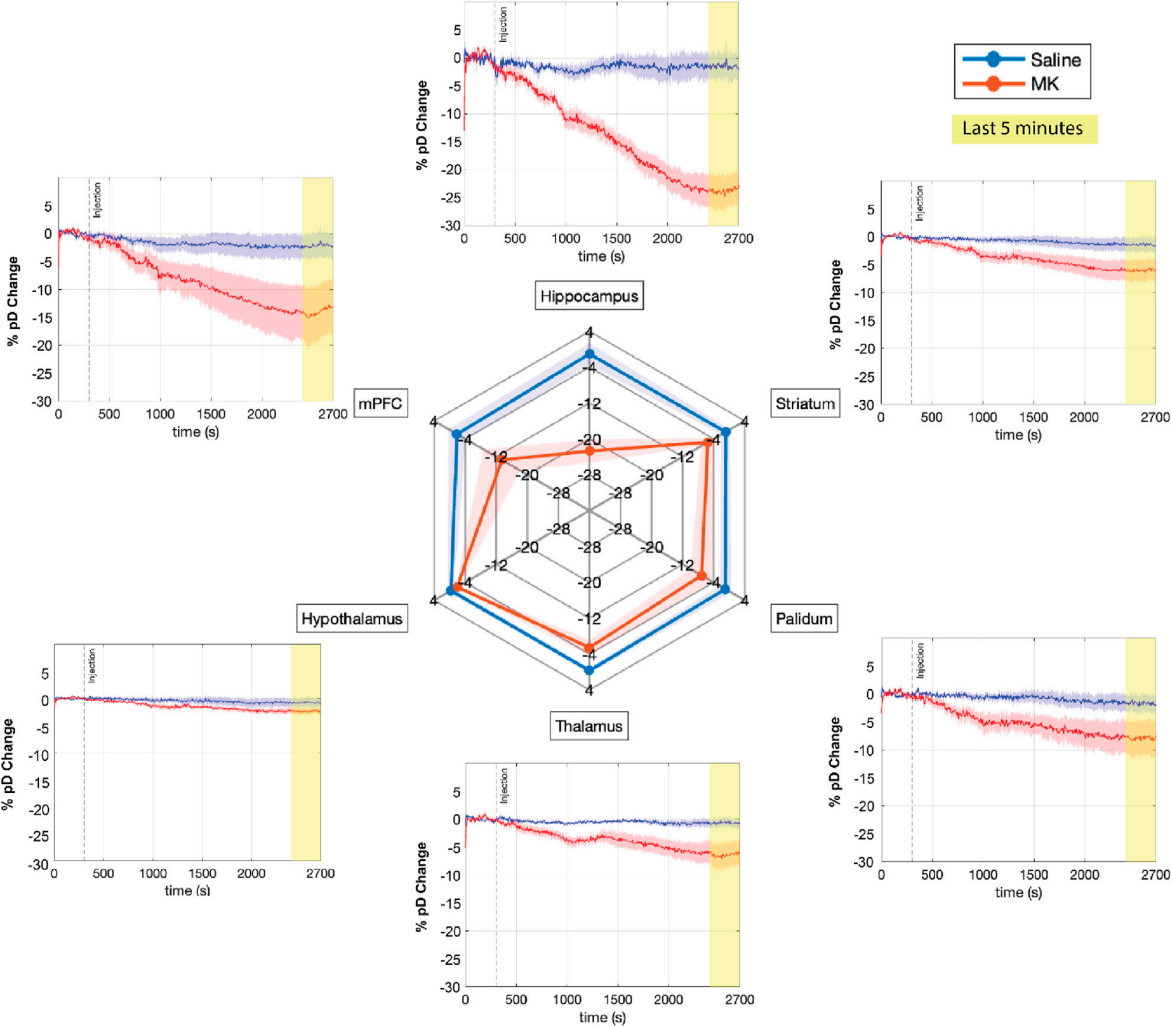
Effects of saline and MK-801 administration on percent CBV changes (% pD change) across multiple brain regions. The time courses of mean percent CBV changes across six ROIs are shown for saline (blue) and MK-801 (red) groups (n = 8 mice per group). The shaded areas represent the standard error of the mean. Time is displayed in seconds, with the injection (saline or MK-801) occurring at t = 300s (dashed vertical line). The last 5 minutes of recording, highlighted in yellow, were used for the spider plot analysis. The spider plot summarizes the mean % pD change during the final 5 minutes for each ROI in both groups.

Consistent with our previous findings Crown et al. (2024), MK-801 administration significantly reduced CBV across multiple ROIs within 40 min after injection compared to saline-treated controls, with the hippocampus showing the most pronounced decrease (Figure 4). To quantify these observations, we conducted a three-way mixed ANOVA with treatment (MK-801 vs. saline) as between-subjects factor, and time (first 5 min vs. last 5 min) and ROIs as the within-subjects factors. The analysis revealed significant main effects for all factors: treatment ( $F_{1,84}=40.515$ , $p=9.836\times 10^{-9}$ ), ROI ( $F_{5,84}=5.691$ , p=0.00014 ), and time ( $F_{1,84}=69.459$ , $p=1.296\times 10^{-12}$ ). Moreover, we observed significant two-way interactions between treatment and time ( $F_{1,84}=17.997$ , p<0.0001 ), ROI and time ( $F_{5,84}=4.0395$ , p=0.0024 ), and treatment and ROI ( $F_{5,84}=3.307$ , p=0.0089 ). Notably, there was a significant three-way interaction among treatment, ROI, and time ( $F_{5,84}=3.307$ , p=0.0089 ). These results indicate that MK-801 not only influenced different brain regions to varying extents but also that the progression of these effects over the 40-min post-injection period varied significantly across regions.

To further elucidate the magnitude of the drug effects across different ROIs, we performed pairwise t-tests comparing pre-injection (initial 5 min) and post-injection (final 5 min) periods for each ROI in both treatment groups. This analysis revealed that MK-801 administration led to significant decreases in Δ CBV across all examined brain regions (p≤0.0406) , with the hippocampus showing the strongest response ( ∼ 23% decrease, Figure 4, spider plot), consistent with its known sensitivity to NMDAR antagonism (Li et al., 2016; Neuhäusel and Gerevich, 2024). The mPFC also exhibited notable changes ( ∼ 13% decrease), suggesting that NMDAR antagonism has far-reaching effects on higher-order cognitive processes. In contrast, saline-treated animals showed no significant changes in any ROI, confirming the specificity of the observed effects to MK-801 treatment.

### 3.2 Functional connectivity analysis reveals differential effects of MK-801 on brain network dynamics

**FIGURE 5:**
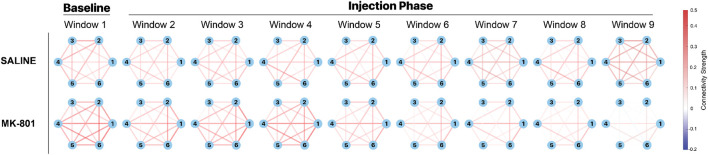
Rolling functional connectivity (RFC) in saline and MK-801 groups of animals. The recorded period is divided into nine non-overlapping 5-minute windows, showing baseline (Window 1, pre-injection) and post-injection dynamics (Windows 2-9). Network diagrams display nodes representing six brain regions: hippocampus (1), striatum (2), pallidum (3), thalamus (4), hypothalamus (5), and medial prefrontal cortex (mPFC) (6). Edges indicate average FC strength across mice using Fisher-transformed Pearson correlation coefficient values. In the saline group (top row), network connectivity remains relatively stable with consistent inter-regional connections throughout the recordings. In contrast, the MK-801 group (bottom row) shows progressive degradation of network connectivity post-injection, resulting in sparse connectivity in later windows. All connectivity values are derived from prewhitened time series data.

Following the prewhitening procedure, we examined the temporal evolution of FC by segmenting the 45-min recording period into distinct temporal epochs. The recordings were partitioned into nine consecutive 5-min windows, with the first window representing baseline activity and windows 2-9 capturing post-injection dynamics (Figure 5). This analysis revealed striking differences in network reorganization between saline and MK-801 groups. In the saline group, we observed relatively stable connectivity patterns across the recordings characterized by moderate to strong connections between most ROI pairs (indicated by red edges). Although some fluctuations were observed, the overall network architecture was largely maintained throughout the recording period. In contrast, the MK-801 group exhibited a markedly different temporal progression. During the baseline period (Window 1), the connectivity patterns exhibited strengths comparable to those observed in the saline group. However, a progressive and systematic degradation of network connectivity became apparent following MK-801 administration. The deterioration was most pronounced at Windows 5-6 (25–30 min post-injection), characterized by a strong reduction in both the number and strength of connections between ROIs. By Windows 7-8 (35–40 min post-injection), the network underwent a dramatic transformation, with most connections substantially weakened and only sparse, isolated connections remaining between particular pairs of ROIs. The divergence between groups became most striking in the final temporal window (40–45 min post-injection). While the saline group maintained a relatively robust and interconnected network structure, the MK-801 group displayed a disrupted connectivity landscape, with minimal interactions between ROIs. This progressive desynchronization of the network suggests that the impact of MK-801 on brain connectivity is not an immediate event but rather a time-dependent process. The gradual nature of this connectivity breakdown potentially reflects the progressive disruption of NMDA receptor-dependent synaptic processes across neural networks, illustrating the complex and temporally nuanced effects of NMDAR antagonism on brain functional architecture.

**FIGURE 6:**
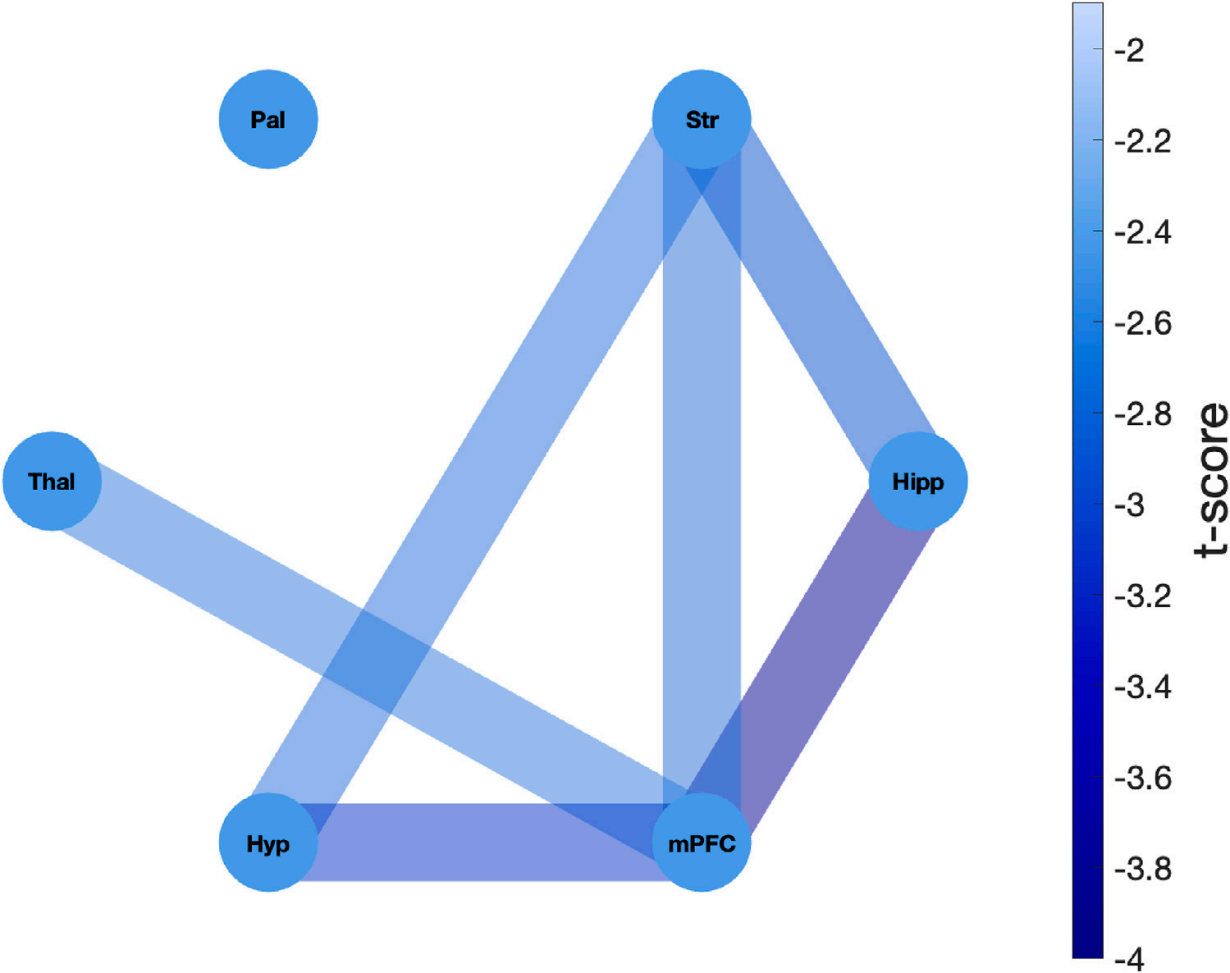
Network representation of differential functional connectivity changes between MK-801 and saline treatments. The network depicts the t-values derived from two-sample independent t-tests directly comparing the distributions of connectivity change slopes between MK-801 and saline groups. For each ROI pair, the t-test evaluated whether the rate of connectivity degradation over time differed significantly between treatment groups (8 slopes per group, one per animal). The network consists of six nodes, each representing an ROI: hippocampus (Hipp), striatum (Str), pallidum (Pal), thalamus (Thal), hypothalamus (Hyp), and medial prefrontal cortex (mPFC). Edges represent the t-statistics, with darker blue edge colors indicating more negative t-values and thus stronger reduction of functional connectivity across time in the MK-801 group compared to the saline group. All observed t-scores were negative, with significant differences observed in hippocampus-mPFC (t = -3.86), mPFC-hypothalamus (t = -3.12), hippocampus-striatum (t = -2.95), striatum-hypothalamus (t = -2.73), striatum-mPFC (t = -2.70), and thalamus-mPFC (t = -2.64) connections.

To systematically assess the differential effects of MK-801 on network organization compared to the saline control, we examined the temporal dynamics of functional connectivity across treatment groups. For each ROI pair and each animal (N = 8 per group), we quantified the rate of change of the functional connectivity by computing the slope of RFC using linear regression. We then compared the resulting slope distributions between the MK-801 and saline groups using two-sample independent t-tests to evaluate whether the rate of connectivity change over time differed significantly between the two groups. The t-score provides a standardized measure of the differences in connectivity trajectories between the two groups. Based on previous findings (Figure 5), we anticipated a reduction in functional connectivity following MK-801 administration. Therefore, we compared the MK-801 group to the saline group, interpreting negative t-scores as indicating a greater connectivity decrease in the MK-801 group (Figure 6).

The analysis revealed profound differences in network connectivity dynamics between the MK-801 and saline groups. All t-scores were negative, indicating a greater reduction in functional connectivity over time in the MK-801 group compared to the saline group. The most pronounced treatment-induced differences in connectivity degradation were observed in pathways involving the hippocampus, mPFC, and striatum. Specifically, the strongest differential effects were detected in connections between the hippocampus and mPFC (t-score = −3.86), hypothalamus and mPFC (t-score = −3.12), and hippocampus and striatum (t-score = −2.95).

Notably, the mPFC exhibited consistently strong decoupling across all circuits in the MK-801 group, except for the pallidum. The most pronounced treatment-related differences (i.e., MK-801 vs. saline) were observed in the reduction of connectivity with the hippocampus (t-score = −3.86). Similarly, the striatum showed a significantly steeper decline in connectivity following MK-801 administration compared to saline group across multiple pathways, including those involving the hippocampus (t-score = −2.95), hypothalamus (t-score = −2.73) and mPFC (t-score = −2.70). These findings provide direct statistical evidence that MK-801 administration induces a significantly greater and progressive disruption of functional connectivity compared to saline, with particularly pronounced effects on limbic-cortical circuits involved in cognitive processing.

On the other hand, some interactions showed less pronounced differences between the two groups, suggesting a relative resilience to the influence of MK-801. In particular, the pallidum exhibited notable stability, with its connections to other brain regions showing no statistically significant differences between the two animal groups. Similarly, the thalamus showed relative resistance to MK-801, with only its connection to the mPFC exhibiting significant changes following MK-801 administration (t = −2.64). This pattern of selective vulnerability offers key insights into the circuit-specific effects of NMDAR antagonism, notably disrupting connectivity in hippocampal and prefrontal regions essential for learning, memory, and executive function, while largely preserving pallidal- and thalamic-based circuit.

## 4 Discussion

The current study offers significant insights into the effects of MK-801 on brain functional connectivity using fUSI technology. By recording high-resolution functional images from anesthetized mice and applying a prewhitening technique to eliminate spurious correlations, we investigated the time-dependent reorganization of neural networks following MK-801 administration. The results indicated that MK-801 disrupts functional connectivity across multiple brain regions, primarily affecting a network involving the mPFC, hippocampus, and striatum. These findings enhance our understanding of how NMDAR hypofunction alters brain-wide connectivity, particularly within circuits associated with cognitive disorders that affect memory and learning.

### 4.1 MK-801 causes reduction of CBV signal

Our results showed that MK-801 administration caused a significant reduction in CBV across multiple regions, with the hippocampus and mPFC showing the most pronounced effects. These findings align with previous studies that have reported hippocampus and mPFC as particularly sensitive to NMDAR antagonism, given their reliance on NMDA receptor-mediated synaptic transmission for proper functioning (Li et al., 2016; Neuhäusel and Gerevich, 2024; Crown et al., 2024). The pronounced impact on the hippocampus may reflect its high density of NMDAR expression, which are crucial for synaptic plasticity and memory formation Yang et al. (2022). This contrasts with regions like the thalamus and pallidum, where differences in receptor density and synaptic architecture may lead to more subtle responses to NMDAR antagonists. Additionally, the significant CBV reduction observed in mPFC is also indicative of its critical role in integrating information across cortical and subcortical regions, processes that rely heavily on NMDAR activity Wang et al. (2013).

### 4.2 MK-801 induces time-dependent network disruption

In addition to regional brain activity reduction, the main finding in our study is that MK-801 administration disrupted the functional connectivity across brain regions. Statistical comparison between treatment groups revealed that MK-801 administration consistently induced significantly greater degradation of functional connectivity over time compared to saline, with the most pronounced effects observed among pathways involving the hippocampus, mPFC, and striatum. The hippocampus-mPFC pathway, which showed the strongest treatment-induced disruption, plays a crucial role in integrating memory with executive control, facilitating top-down regulation and cognitive flexibility (Preston and Eichenbaum, 2013; Friedman and Robbins, 2022; Malik et al., 2022). Studies have shown that the mPFC interacts with hippocampus for rapid learning and memory consolidation, supporting decision-making processes that require recalling appropriate actions or emotional responses in specific contexts Euston et al. (2012). The significant differential disruption of the hippocampus-mPFC pathway can therefore explain memory and learning impairments commonly observed in NMDAR dysfunction models.

Additionally, the striatum, which has a key role in reward processing Cox and Witten (2019), habit formation Yin and Knowlton (2006) and action selection Markowitz et al. (2018), interacts with the mPFC Howland et al. (2022); Wilhelm et al. (2023) and hippocampus Ross et al. (2011). The hippocampus-striatum pathway supports the integration of spatial and contextual memory with reward-based behavior Ross et al. (2011), while the mPFC-striatum connection enables the regulation of goal-directed versus habitual actions (Corbit, 2018; Balleine and O’Doherty, 2010). The disruption in striatum connectivity, particularly with the hippocampus and mPFC can impair goal-directed behavior, memory, decision-making process, and cognitive flexibility, leading to maladaptive behaviors often observed in neuropsychiatric disorders such as addiction, schizophrenia, and obsessive-compulsive disorder (Saxena et al., 1998; Graybiel and Rauch, 2000; Sigurdsson and Duvarci, 2016; Meyer-Lindenberg et al., 2002; Benetti et al., 2009; Henseler et al., 2010).

Interestingly, pallidum-based circuits exhibited remarkable resilience to MK-801 administration, showing no significant differences in functional connectivity changes compared to saline administration. This pattern of selective resilience aligns with previous research indicating that the globus pallidus exhibits a minimal metabolic response to NMDAR antagonists compared to other brain regions Miyamoto et al. (2000). Such regional specificity may be attributed to differences in NMDAR subunit composition, receptor density, or the unique architecture of pallidal circuitry, suggesting that NMDAR antagonism impacts neural networks in a heterogeneous manner.

While our study predominantly identified time dependent MK-801-induced decreases in functional connectivity between key regions such as the hippocampus, mPFC, and striatum, other studies reported diverse findings regarding circuit-specific connectivity alterations in schizophrenia models. Recent multi-modal analysis of NMDAR dysfunction in schizophrenia has reported increased connectivity between striato-pallido-thalamic and cortical regions of the auditory-sensory-motor network Gaebler et al. (2023). Furthermore, other NMDAR antagonist agents such as Traxoprodil, have been found to increase hippocampal-prefrontal coupling Becker et al. (2019), while ketamine has been shown to enhance functional connectivity of the ventral striatum/nucleus accumbens and ventromedial prefrontal cortex Dandash et al. (2015). Conversely, other studies, along with ours, have observed impairements in the network, including reduced hippocampal functional connectivity and decoupling of the medial temporal, sensorimotor, frontoparietal and lateral-temporal networks (Samudra et al., 2015; Peer et al., 2017).

These apparent discrepancies may reflect important differences in species-specific network organization, the effects of acute versus chronic NMDAR hypofunction, variations in the dose and type of agents used, or differences in methodological approaches to connectivity analysis. Importantly, the high dose of MK-801 employed in our study, along with pharmacological differences between the various NMDAR antagonists and modulators used in other studies, may be particularly influential factors. Notably, the implementation of prewhitening techniques to address non-stationarity may reveal functional relationships obscured in traditional correlation analyses. Furthermore, the opposite directionality of connectivity changes may be network-specific, wherein NMDAR antagonism disrupts the excitatory/inhibitory balance differently across distinct neural circuits. These complementary findings suggest that NMDAR hypofunction does not produce uniform effects across brain networks but instead triggers a complex interplay of hyper- and hypo-connectivity, collectively contributing to the cognitive impairments observed in neuropsychiatric disorders.

### 4.3 Genetic insights into NMDAR dysfunction in neurological and psychiatric disorders

Recent advances have significantly enhanced our understanding of the NMDAR hypothesis in a range of neurological and psychiatric disorders Mota Vieira et al. (2020). This hypothesis has gained considerable support through the identification of both common and rare genetic variants associated with the NMDAR signaling pathway. Notably, an increasing number of variants in the GRIN genes, which encode subunits of the NMDAR, have been identified in patients with diverse neurological and psychiatric conditions, including autism spectrum disorder, epilepsy, intellectual disability, attention-deficit/hyperactivity disorder, and schizophrenia (Platzer and Lemke, 2018; Myers et al., 2019). Among these, the GRIN2A gene, encoding the GluN2A subunit of the NMDAR, has emerged as a significant contributor to disorders such as schizophrenia (Singh et al., 2022; Trubetskoy et al., 2022) and the epilepsy spectrum (Lemke et al., 2013; Samanta, 2023). The Allen Mouse Brain Atlas reveals pronounced GRIN2A expression in both the hippocampal formation and isocortex, particularly in medial prefrontal regions. This can explain the increased sensitivity of these regions to NMDAR antagonism observed in our study, where the hippocampus and mPFC exhibited the most substantial CBV and functional connectivity reduction. These genetic findings complement and extend pharmacological models such as the one employed in our study, providing converging evidence for NMDAR dysfunction as a central mechanism in a broad range of neurological and psychiatric disorders.

### 4.4 The importance for prewhitening the power doppler time series

Functional connectivity studies often rely on correlating time series data that have undergone various preprocessing steps, such as filtering, adjustment, smoothing, and averaging. However, an important aspect of time series data, such as their internal structure - specifically nonstationarity and autocorrelation - has not always been fully considered. When correlations are calculated without accounting for these factors, there is a risk that the resulting values may reflect a mix of unrelated influences, such as the intrinsic properties of the time series, external factors, and the true relationship between the two series, rather than providing an accurate representation of their actual association Haugh (1976). It is worth quoting from Granger and Newbold’s study Granger and Newbold (1974) in 1974: “*We find it very curious that whereas virtually every textbook on econometric methodology contains explicit warnings of the dangers of autocorrelated errors, this phenomenon crops up so frequently in well-respected applied work*” (Granger and Newbold, 1974, p. 111). This observation points out the persistent importance of properly addressing autocorrelation in time series analysis.

A key contribution of the current study is the introduction of a prewhitening approach to fUSI data analysis, which transforms the recorded pD signal into a stationary process by removing time-dependent trends and periodicities. We show that the raw pD time series exhibit non-stationarities that, if unaddressed, can lead to spurious correlations. By implementing prewhitening, we mitigate these issues, ensuring a more accurate assessment of pairwise FC analysis. This is particularly important for studying dynamic brain connectivity, where temporal fluctuations can confound results. Our analysis shows that prewhitening improves the reliability of FC metrics, with a significant reduction in spurious correlations compared to non-prewhitened data. This methodological advance demonstrates the necessity of preprocessing steps like prewhitening when investigating FC in both experimental and clinical settings, offering a robust solution to the challenges posed by non-stationary pD signals.

### 4.5 Limitations of the current study and future directions

One potential limitation of our study is the use of isoflurane anesthesia, which is a known vasodilator that can impact cerebral blood volume (CBV) and flow (CBF) (Masamoto and Kanno, 2012; Franceschini et al., 2010). Isoflurane also modulates NMDA and GABA receptors McAuliffe et al. (2009), potentially interacting with the effects of MK-801 on NMDAR function. Previous studies have shown that isoflurane can alter functional connectivity patterns, particularly in thalamocortical and cortico-cortical connections, with these effects varying based on anesthesia depth (Grandjean et al., 2014; Bukhari et al., 2017). However, since both control and MK-801-treated groups underwent identical anesthesia protocols, the between-group differences in our study can primarily be attributed to MK-801 administration. Additionally, since our study was conducted in anesthetized animals, we were unable to observe the behavioral effects of MK-801 and directly link them to changes in brain activity and connectivity. Given that MK-801 affects regions involved in memory and learning, future studies should involve awake, behaving animals performing memory- and learning-associated tasks, such as novel object recognition and the Barnes Maze. These studies should incorporate fUSI recordings to correlate behavioral changes with brain activity and connectivity alterations. Additionally, a future awake-behaving experiment would also be valuable in isolating the specific effects of MK-801 from those potentially influenced by anesthesia. Furthermore, while we explored connectivity changes in key brain regions, such as the hippocampus and mPFC, we were unable to assess the potential effects of MK-801 administration on other regions within the septohippocampal network, including the nucleus accumbens (NAc), amygdala, and medial septal nucleus (MSN), as these areas were not accessible in the selected sagittal 2-dimensional imaging plane. Future studies will involve the newly developed 3-dimensional ultrasonic probes, such as matrix arrays and raw column arrays (RCA) (Rabut et al., 2019; Sauvage et al., 2020; Bertolo et al., 2021) to generate volumetric images of the mouse brain providing access to all areas of the septohippocampal network. Furthermore, the current study implements only a single-dose protocol of MK-801, which may not fully capture the dose-dependent effects of the agent. Given the established dose-dependent effects of MK-801 on hippocampal theta and gamma oscillations Saunders et al. (2012), it is important for future studies to evaluate how different doses affect CBV and functional connectivity in the recorded brain areas. Overall, despite the limitations, our study provides direct evidence that MK-801 disrupts connectivity between key brain regions involved in learning, memory and other higher-order cognitive functions. These findings offer valuable insights into the neural basis of cognitive impairments associated with various neuropsychiatric disorders and open new avenues for future research into targeted interventions.

## Data Availability

The original contributions presented in the study are included in the article/supplementary material, further inquiries can be directed to the corresponding author.
